# ViR: a tool to solve intrasample variability in the prediction of viral integration sites using whole genome sequencing data

**DOI:** 10.1186/s12859-021-03980-5

**Published:** 2021-02-04

**Authors:** Elisa Pischedda, Cristina Crava, Martina Carlassara, Susanna Zucca, Leila Gasmi, Mariangela Bonizzoni

**Affiliations:** 1 Department of Biology and Biotechnology, University of Pavia, 27100 Pavia, Italy; 2 ERI BIOTECMED, Universitat de Valencia, 46010 Valencia, Spain; 3 Engenome Srl, 27100 Pavia, Italy

**Keywords:** Viral integration, Repetitive DNA, Lateral gene transfer, Non-model organisms

## Abstract

**Background:**

Several bioinformatics pipelines have been developed to detect sequences from viruses that integrate into the human genome because of the health relevance of these integrations, such as in the persistence of viral infection and/or in generating genotoxic effects, often progressing into cancer. Recent genomics and metagenomics analyses have shown that viruses also integrate into the genome of non-model organisms (i.e., arthropods, fish, plants, vertebrates). However, rarely studies of endogenous viral elements (EVEs) in non-model organisms have gone beyond their characterization from reference genome assemblies. In non-model organisms, we lack a thorough understanding of the widespread occurrence of EVEs and their biological relevance, apart from sporadic cases which nevertheless point to significant roles of EVEs in immunity and regulation of expression. The concomitance of repetitive DNA, duplications and/or assembly fragmentations in a genome sequence and intrasample variability in whole-genome sequencing (WGS) data could determine misalignments when mapping data to a genome assembly. This phenomenon hinders our ability to properly identify integration sites.

**Results:**

To fill this gap, we developed ViR, a pipeline which solves the dispersion of reads due to intrasample variability in sequencing data from both single and pooled DNA samples thus ameliorating the detection of integration sites*.* We tested ViR to work with both in silico and real sequencing data from a non-model organism, the arboviral vector *Aedes albopictus.* Potential viral integrations predicted by ViR were molecularly validated supporting the accuracy of ViR results.

**Conclusion:**

ViR will open new venues to explore the biology of EVEs, especially in non-model organisms. Importantly, while we generated ViR with the identification of EVEs in mind, its application can be extended to detect any lateral transfer event providing an ad-hoc sequence to interrogate.

## Background

The transfer of genetic material between separate evolutionary lineages is a recognized event that occurs not only among prokaryotes, but also between viruses and eukaryotic cells [1]. Somatic integrations of different viral species, among the best of known of which are the human papilloma virus, hepatitis B and C viruses and the Epstein-Barr virus, have been linked to genotoxic effects possibly progressing into cancer [2]. Consequently, several pipelines have been developed to identify viral sequences integrated into the human genome using whole-genome sequencing (WGS) data (i.e. HIVID, SummonChimera, Vy-PER; HGT-ID, ViFi, VirTect, BS-virus-finder, Seeksv) [3–10]. Each of these computational methods is versatile in terms of data input format (e.g., RNA-seq, DNA-seq or bisulfite sequencing data), reference viral databases or customization opportunities, sensitivity and accuracy and CPU requirements. All these pipelines have in common the fact that they were designed for the well-annotated human genome. Viruses can also integrate into the genome of germline cells. The persistence and the outcome of these integrations, which are vertically transmitted, depend on their effects on the fitness of the host [11]. The existence of these Endogenous Viral Elements (EVEs) has long been known, with studies focusing mainly on EVEs from retroviruses in mammalian genomes [12, 13]. The recent development of modern genomic sequencing approaches has opened to the study of non-model organisms. The genomes of organisms as different as arthropods, fish, snakes, birds, vertebrates and plants were shown to host EVEs, which derive not only from DNA viruses and retroviruses, but also from nonretroviral RNA viruses [14–22]. In these non-model organisms, EVEs range widely in numbers and tend to occur in repetitive DNA, mostly in association with transposable element (TE) sequences [20, 23, 24]. EVEs of non-model organisms have been increasingly recognized as important players in different biological processes such as antiviral immunity and regulation of expression [25, 26]. However, rarely studies of EVEs in non-model organisms have gone beyond their characterization from reference genome assemblies. The lack of bioinformatic tools able to account for repetitive DNA when mapping WGS data to a genome sequence is hindering our ability to detect integration sites different than those already annotated in the assembly. As a consequence, it is difficult to understand the widespread occurrence and polymorphism of EVEs using WGS data from wild-collected samples and testing hypothesis on EVE function using WGS data from samples collected under hypothesis-driven experimental conditions. To ameliorate this issue, we have developed a new bioinformatic pipeline, ViR. ViR works downstream of any currently available EVE prediction tool using paired-end reads to improve the characterization of integration sites by solving the dispersion of reads in genome sequences that are rich of repetitive DNA. We tested ViR with low- and high-coverage WGS data from *Aedes albopictus,* to date the mosquito species with the largest genome size and highest TE content among Culicinae [27, 28]*,* followed by molecular validation of the predicted insertion sites. Additionally, the performance of ViR was tested using in silico WGS data*.*

Importantly, ViR can be adopted to interrogate any genome for the presence of non-host sequences, showing its applicability beyond the identification of viral integration sites and facilitating studies of lateral gene transfer (LT).

## Results

### Overview of the ViR pipeline

**Fig. 1:**
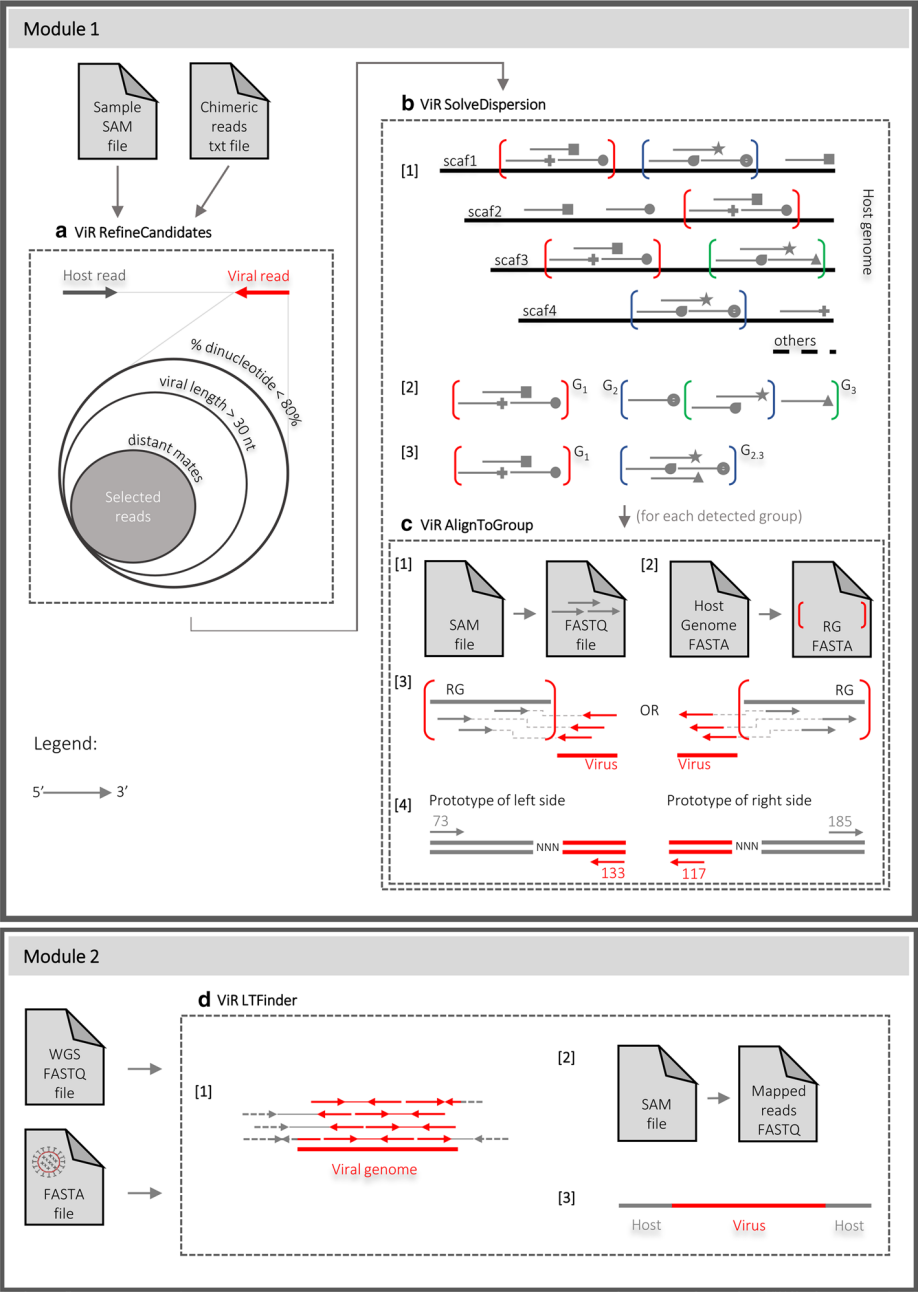
Overview of ViR. ViR comprises four scripts, organized into two modules. Module 1 starts with the ViR_RefineCandidate script, which uses the SAM file of the WGS data alignment to the host genome and a file with the list of chimeric reads. **a** ViR_RefineCandidates filters the viral mate of the chimeric reads pairs to identify the best candidate viral reads. In chimeric reads, the host read is shown in in gray, the viral read, in red. Flags of alignment and sequence quality information are extracted for the identified best candidates. The output directory of ViR_Refine Candidates is the input of ViR_SolveDispersion. **b** ViR_SolveDispersion is designed to identify groups of host reads supporting a potential integration site in equivalent genomic regions (step 1). Read groups are compared in a pair-wise mode to merge groups sharing a certain percentage of reads (step 2). Remaining read groups support potential viral integrations (step 3). The usage of ViR_AlignToGroup script is embedded in ViR_SolveDispersion. **c** For each identified read group ViR_AlignToGroup extracts the reads from the SAM file of the selected reads by ViR_RefineCandidates (step 1) and the sequence of the equivalent region of the group in FASTA format (step 2). Then, reads are re-aligned to the sequence of the equivalent region and flags of alignment are used to identify the left and right side of the integration site (step 3). Examples of flags supporting the right (i.e., 73, 133) and left (i.e., 117, 185) ends of the integration site are shown (step 4). All the reads are represented by arrows with standard or not standard terminal part. The direction of the arrow is 5′–3′. In Module 2 the FASTQ file of the sample WGS and the FASTA file of the viral genome(s) are the input for ViR_LTFinder script. **d** ViR_LTFinder align WGS raw reads to the viral genome(s) (step 1). Aligned reads are extracted, converted into FASTQ (step 2) and used for local *de-novo* assembly (step 3). Aligned reads include mates in which both reads map to the viral genome (in red), chimeric reads (viral read in read and host read in dashed grey) and mates in which one is a soft clipped read (viral portion in red, the rest in dashed gray). Mates are indicated by continuous thin line

Any of the currently available tools to identify viral integrations from paired-end sequencing data operates by selecting chimeric pairs, in which one read maps to the host genome (the host read) and the other to the viral genome (the viral read) [29]. If the integration site occurs in a repeated region, host reads may potentially map to all the regions in which this repeat occurs in the reference genome. As a consequence, host read supporting a viral integration, will distribute across these “equivalent” mapping genomic positions, and the signal for the integration site, expressed in terms of host reads coverage, may not reach the threshold of detection. This situation is exacerbated in non-model organism, with genome assemblies in which a sequence may have been assembled into different contigs or scaffolds and that are rich of repetitive DNA. To ameliorate the prediction of viral integration sites, we developed ViR, a pipeline composed of four scripts divided into two modules (Fig. 1).

The first script of the pipeline is ViR_RefineCandidates which requires two input files: the SAM file of the WGS data alignment to the host genome and a file with chimeric reads. The script identifies the “best candidate” pair of reads supporting a potential viral integration through subsequent filtering steps (Fig. 1a). The output directory of ViR_RefineCandidates is used as input of the next script, ViR_SolveDispersion. Using the script ViR_SolveDispersion, host reads that map to “equivalent” genomic positions are grouped together (Fig. 1b). Each group includes chimeric reads in which the host reads map to equivalent regions and the viral reads map to the same viral species. Additional information on these regions can be collected by providing input files with custom data (i.e., mapping coordinates of EVEs annotated in the reference genome, of TEs, etc.). This additional information is useful to distinguish between polymorphisms of EVEs *versus* new integrations from the same virus. ViR_SolveDispersion includes the usage of the script ViR_AlignToGroup, which for each detected group, extract the sequence of the equivalent genomic region and re-align host reads to it. This procedure allows to predict the left and right sides of the integration site through the use of alignment flags (Fig. 1c).

ViR comprises an additional script, ViR_LTFinder, which runs independently from the others (module 2). This script uses WGS data to map against a non-host sequence (i.e. entire genomes, genes, transposable elements, viruses). Aligned reads are extracted and used for a de novo assembly using Trinity. Resulting assembly/ies may include the consensus sequence for the LT event (Fig. 1d).

### Implementation of ViR with WGS data from *Aedes albopictus*

ViR was run using WGS data from wild-collected *Ae. albopictus* mosquitoes. *Aedes albopictus* has the largest mosquito genome sequenced to date, the majority of which is repetitive DNA [27, 28]. For *Ae. albopictus*, three genome assemblies are available, which differ greatly in the number of scaffolds and are all larger than the expected size of the genome based on cytofluorimetric estimates, suggesting duplications [27, 28, 30]. We selected to run ViR using two assemblies, AaloF1 and AalbF2, which have 154,782 and 2197 scaffolds, respectively. Hundreds of EVEs from various taxonomic viral categories are annotated in AaloF1 and AalbF2 [23, 28], giving further complexity in the identification of novel integration sites from WGS of wild-collected samples, especially considering that integration events are expected to be rare and in repetitive DNA [25].

**Fig. 2:**
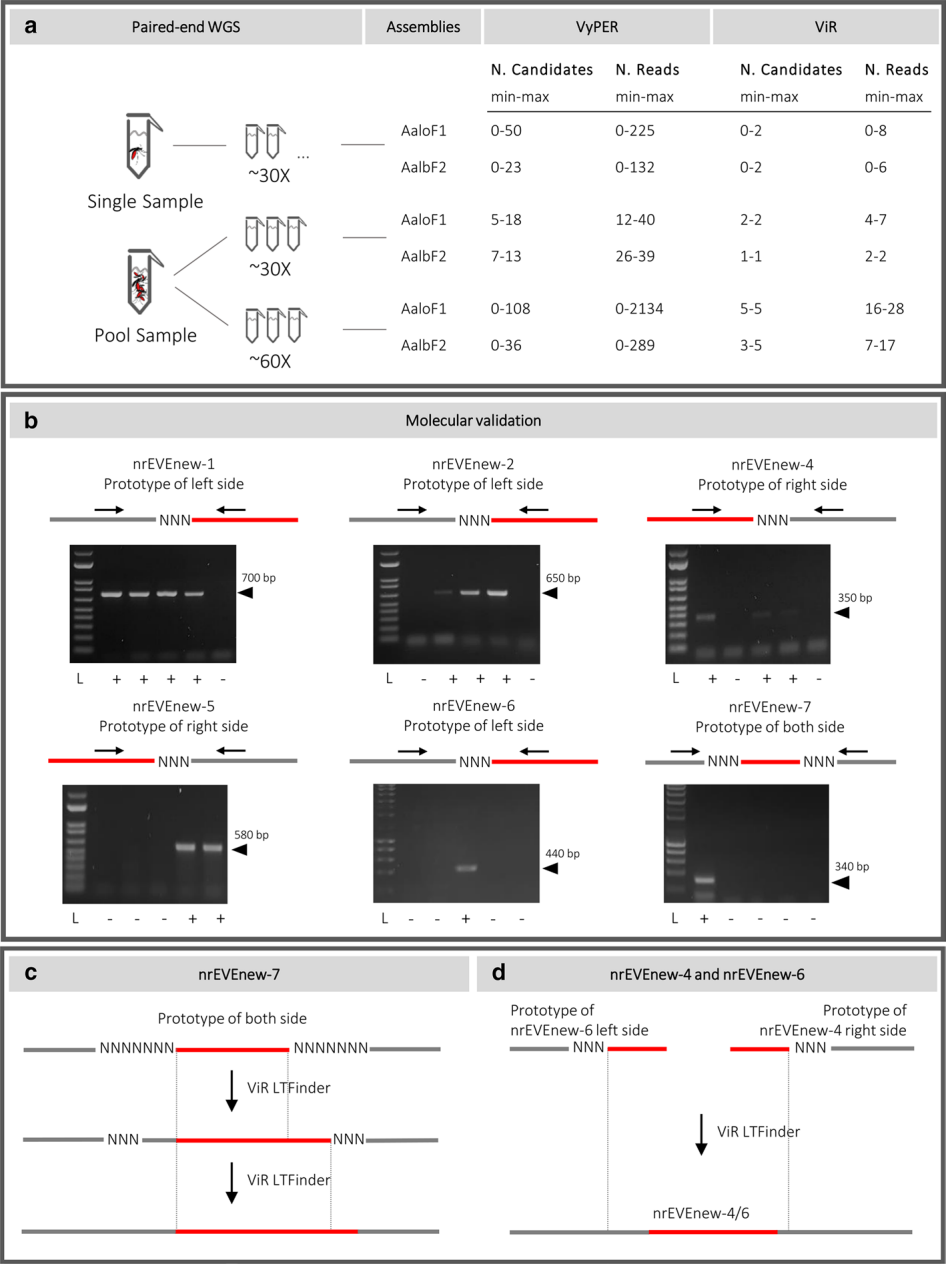
Implementation of ViR with WGS data from *Ae. albopictus*. **a** ViR was run with WGS data generated from DNA of both single and pool DNA samples using two assemblies, AaloF1 and AalbF2 [27, 28] and a custom database of viral genomes (Additional file 1). A list of chimeric pairs, indicative of potential integration sites, were obtained for each sample running Vy-PER [5]. Running Module 1 using the chimeric reads identified by Vy-PER resulted in a total of seven potential viral integrations. **b** Scheme of potential viral integrations and their molecular validation. Each lane is the result of a PCR on mosquito genomic DNA with primers that were designed to check the left or right integration sites. ‘+’ indicates the presence of the viral integration and ‘−’ the absence. **c** Identification of exact integration sites of nrEVEnew-7 running ViR_LTFinder; **d** ViR_LTFinder was run with WGS data and the viral sequences of nrEVEnew-4 and nrEVEnew-6 showing that they correspond respectively to the right and left regions of the same integration, which was molecularly validated. ‘+’ indicates the presence of the viral integration and ‘−’ the absence

We generated WGS data from DNA of both single and pooled samples and at different coverage to compare the results of ViR across different conditions (Fig. 2a). Single sample mosquitoes (SSMs) consisted of twenty-two mosquitoes whose genomes were analyzed independently [31]. Pool samples were 6 samples consisting each of the DNA from 30 mosquitoes. Three samples were sequenced at an approximate coverage of 30 × and called pool30; the remaining samples were sequenced at an approximate coverage of 60 × and called pool60. A list of chimeric pairs, indicative of potential integration sites, were obtained for each sample running Vy-PER [5] with a custom-made database of viral genomes (Additional file 1). The number of chimeric reads identified by VyPER ranges from 0 to 2134 (Additional file 2); these host read of these pairs are spread across different regions of the host genomes (Fig. 2a) and viral reads include homopolymers or low-complexity sequences.

#### Testing module 1

ViR_RefineCandidates and ViR_SolveDispersion were run with standard parameters for all samples using both AaloF1 and AalbF2 (Fig. 2a). Publicly available annotations of EVEs and TEs were used to define the genomic context of each equivalent region [23, 28].

Overall, across all samples, a total of seven integrations, all of viruses from the Flaviviridae family were identified, with a different support of reads (Fig. 2a). Both the right and left sides of the integration were resolved bioinformatically for nrEVEnew-7. Only the left or right sides of the integration were bioinformatically predicted from nrEVEnew-1, nrEVEnew-2 and nrEVEnew-6 or nrEVEnew-4, nrEVEnew-5 and nrEVEnew-8, respectively. nrEVEnew-2, nrEVEnew-4, nrEVEnew-6 and nrEVEnew-7 were detected when running ViR using both AloF1 and AalbF2; nrEVEnew-1, nrEVEnew-5 and nrEVEnew-8 were detected only when running ViR with AalbF2, possibly because of the higher completeness of AalbF2 [28] versus AaloF1 [27]. These predictions were molecularly validated, with the exception of nrEVEnew-8 (Fig. 2b). The presence of nrEVE-new-8 is supported by three reads in one of the pool60 samples. As a consequence, we cannot exclude that the absence of amplification of nrEVE-new-8 in the tested samples, is not due to its rarity and/or population-specific occurrence, as observed in *Ae. aegypti* [25].

#### Testing module 2

ViR-LTFinder was designed to detect LT events from sequences with no similarity to host genomes. We reasoned that the above-described novel viral integrations, which can be unambiguously differentiated from EVEs annotated in the reference genome assembly, are novel LT events. Thus, for each of the novel viral integration predicted using the scripts of the module 1, we run ViR_LTFinder using as non-host sequence the viral sequence supported by the chimeric reads.

Running ViR_LTFinder with our WGS data and using as anchor the viral portion of nrEVEnew-7 built to a consensus sequence of 1237 bp identifying the left and right side of the integration event (Fig. 2c). ViR_LTFinder was further tested for nrEVEnew-1, nrEVEnew-2, nrEVEnew-4, nrEVEnew-5 and nrEVEnew-6. While our WGS data did not include reads able to solve the right or left integration site for nrEVEnew-1and nrEVE-new-2 or nrEVEnew-5, respectively, results of ViR_LTFinder showed that nrEVEnew-4 and nrEVEnew-6 correspond respectively to the right and left regions of the same integration, including a 534 bp viral sequence. This viral integration was molecularly validated (Fig. 2d). Viral integrations identified running ViR, including both module 1 and module 2, in the genome of *Ae. albopictus* mosquitoes, are described in Table 1.

**Table 1:** Viral integrations identified running ViR with WGS data from *Ae. albopictus* mosquitoes

nrEVE	Length (bp)	Virus (blastx id %)	Viral protein
nrEVEnew-1	295	KRV (70%)	NS1-NS2A
nrEVEnew-2	578	KRV (82%)	NS3
nrEVEnew-4/6	534	AeFV (75%)	NS5
nrEVEnew-5	880	KRV (78%)	NS5
nrEVEnew-7	318	AeFV (91%)	E

### ViR performance in solving dispersion of host reads

**Fig. 3:**
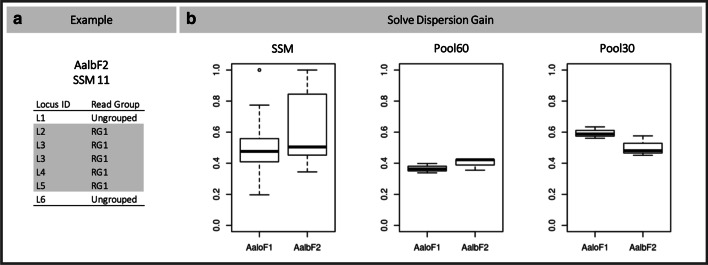
Testing ViR performance in solving read dispersion. **a** Seven pairs of reads were identified by Vy-PER as potential viral integrations in SSM 11. ViR grouped five of these reads into a single group which led to the identification of nrEVEnew-4 and resulting in a Solve Dispersion Gain value of 0.65. **b** Values of Solve Dispersion Gain were calculated for all single and pool samples

We tested the benefits of running ViR by calculating the solve dispersion gain parameter. For each sample, we considered as dataset the whole list of reads resulting from ViR_RefineCandidates. We quantified the gain comparing the host-mapping loci identified by Vy-PER and the read groups created by ViR_SolveDispersion. As an example, in replicate 11 among SSMs, seven pairs of reads were identified by Vy-PER as potential viral integrations. ViR solved the dispersion of these seven reads by grouping them into one group supporting nrEVEnew-4; two reads remained ungrouped, resulting in a Solve Dispersion Gain value of 0.65 (Fig. 3a). In SSMs, the median values of the solve dispersion gain were 0.47 and 0.5 when using AaloF1 and Aalb2, respectively. In pool samples, the median values in pool60 were 0.36 and 0.42 and in pool30 were 0.58 and 0.48 when using AaloF1 and Aalb2, respectively (Fig. 3b). Thus, we noticed a significant gain in using ViR, also in the improved version of the *Ae. albopictus* assembly, AalbF2 [28]. Dispersion gain values were not different between single vs pools or between pools 30 vs pools 60 samples, indicating the gain is not influenced by the sequencing of pools or single samples nor the depth of sequencing.

In each dataset a certain number of chimeric reads identified by Vy-PER could not be grouped. These reads have alignments in the genome distant from the others and cannot be used to predict potential viral integrations. Even if they are not useful for the discovery of viral integrations, it is important to isolate them to avoid wasting time trying to interpret them, for this motif we included these ungrouped reads in the calculation of the Solve Dispersion Gain. These ungrouped reads were more abundant when testing ViR with AaloF1 than AaloF2 as a result of the higher fragmentation of the AaloF1 assembly [28].

### Evaluation of ViR performance using in silico dataset

We tested the performance of ViR with simulated dataset, including samples sequenced singly and in pools (Additional file 3). Performance of module 1 and module 2 were tested separately considering five different sequencing coverage depths and simulating three different integration events in unique genomic loci or repeated genomic regions. For pools, different pool sizes were also analyzed. ViR performance was computed based on the confusion matrix [32], through the following parameters: accuracy, sensitivity, specificity, precision, F1, balanced accuracy and Matthews Correlation Coefficient (MCC) [32, 33]. Overall results are summarized in Additional file 3.

ViR module 1 showed 100% sensitivity and 100% specificity in all cases with SSMs. Across all tested conditions in SSMs, the performance of module 2 reached an overall accuracy and specificity of 93.75% and 78.94%, respectively. This result was driven by the situation in which the integration site occurred in a highly repeated (100 times) genomic sequence, giving a ViR accuracy of 83.33% and a specificity of 55.55%. When using pools, the sensitivity and the accuracy greatly varied with the size of the pool (pools of 50 individuals had accuracy and sensitivity < 50% in both modules), the sequencing coverage (with a 30X coverage accuracy was 55% and 38% in module 1 and 2 respectively, but increased to 72%, in module 1, and 64%, in module 2, for a 60X coverage), the length and the site of the integration event. Integrations shorter than 300 bp will not be able to be sensitively detected.

## Discussion

The purpose of ViR is to provide reliable identification of EVEs using WGS data, especially when viral integrations occur in repetitive regions of host genomes or when using genome assemblies rich in repetitive DNA and/or suffering from fragmentations.

Currently available EVE identification tools are mostly based on the identification of chimeric reads. ViR works with the list of these chimeric reads and ameliorates EVE prediction. The step of improving EVE prediction is important because the advent of next-generation sequencing technologies and metagenomic analyses showed that viral integrations occur in non-model organisms as frequently as in model organisms (i.e. human, mouse and *Dr. melanogaster*) [20, 23–25, 28], but genome assemblies of many non-model organisms are often still fragmented or richer in repetitive DNA [23, 27, 28, 34–36]. Both these elements generate intra-host variability when trying to map short paired-end reads and identifying chimeric reads to predict integration sites. To overcome this limitation, we have developed ViR, a bioinformatic pipeline suited to account for intrasample variability to ameliorate predictions of viral integrations. We evaluated ViR performance using in silico WGS data. In SSMs, ViR always detected viral integrations except for integration events occurring in a highly (> 100 times) repetitive genomic region. This result is probably due to the intra sample variability that affects the generation of de novo assemblies. In pooled sample sequencing, ViR limits are based on the size of the pools and the sequencing depth. A pool of 50 individuals, sequenced at a 30 × coverage will be problematic. Additionally, the site of the integration event will be reliably identified unless it occurs in a highly (> 100 times) repeated genomic sequence and it includes a short (< 300 bp) viral portion.

We further implemented ViR on WGS data from *Ae. albopictus*, the mosquito with the largest genome to date, a TE content > 50% of the genome and two assemblies differing in completeness [27, 28]. In the absence of a true set of viral integrations, ViR performance with real data was tested by calculating the dispersion gain, a parameter from the Information Theory [37] and molecularly validating predicted viral integrations.

We demonstrated that ViR is able to solve the dispersion of reads supporting a viral integration similarly when using WGS data from single and pool samples and with a coverage of between 30 and 60x. We anticipate ViR will open new venues to explore the biology of EVEs, especially in non-model organisms. The design of ViR makes it compatible with any list of putative chimeric reads produced by any currently available EVE identification tool [3–9, 29], giving users great flexibility. Importantly, while we show the applicability of ViR to detect EVEs, this pipeline can be adopted to detect any LT event providing an *ad-hoc* sequence to interrogate.

## Conclusions

In summary, we show that ViR is a robust method to account for intrasample variability when annotating LT events into host genomes in analysis of both real and simulated data. By releasing ViR as an open-source pipeline we aim to provide an accurate tool for interrogating genomes, extending the analyses of LT events to non-model organisms.

## Methods

### Pipeline implementation

The pipeline is composed of four scripts, which work in two modules. The first module includes three scripts, ViR_RefineCandidates, ViR_SolveDispersion and Vir_AlignToGroup, which work together to overcome the dispersion of reads due to intrasample variability. The second module includes one script, ViR_LTFinder, designed to test for LT events of non-host sequences which have none or limited (defined by the user) sequence similarity to sequences of the host.

*ViR_RefineCandidates.* This script selects from a list of chimeric reads the best candidate pairs supporting a viral integration by filtering reads. The filtering criteria include: (1) filter reads based on their sequence complexity, expressed as percentage of dinucleotides (default < 80%); (2) imposing a minimum length recognized as viral (default 30 nucleotides); (3) removing mates that can align within a defined window in the reference genome (default 10,000) based on Blast and BEDtools packages, respectively [38, 39] (Fig. 1a).

Inputs for ViR_RefineCandidates are a text file of paired-end chimeric reads and the SAM file of the reads aligned to the host genome. Paired-end chimeric reads are pairs in which one read maps to the host genome (hereafter called host read) and its pair maps to a virus (hereafter called viral read) [29]. The script is versatile and accepts as input the text file listing chimeric pairs, independently of the used tools. If no pair of reads pass the filtering steps, the script prints a stop message. Otherwise, an output file is generated, which collects information on both the viral and host read (Additional file 4).

*ViR_SolveDispersion.* This script solves the dispersion of host reads by grouping together reads that map to regions of the genome with the same sequence (Fig. 1b). These reads are called “read groups”; regions of the genome to which these reads can equivalently map because they contain the same repetitive element, or it is a sequence that has been erroneously assembled into different contigs or scaffolds, are called “equivalent regions”. The script acquires as inputs a file listing all samples to analyze and the output directory of ViR_RefineCandidates. Reads mapping within equivalent regions are grouped together using the function “merge” from bedtools (Fig. 1b, step 1). The length of the equivalent region is defined by the user by setting the maximum distance among host reads and the minimum number of host reads within each region; defaults for these two parameters are 1000 base pairs (bp) and 2 reads, respectively. Then, identified read groups are compared in a pairwise mode in an iterative process in which read groups sharing more than a user-defined percentage of reads are collapsed in one (default is 80%) (Fig. 1b, step 2). This procedure allows to identify the best candidate anchor genomic region of a potential viral integration site (Fig. 1b, step 3). Options to describe the genomic context of each region are available by proving adequate input files.

*ViR_AlignToGroup.* This script predicts the right and left sides of the potential integration site by realigning reads supporting each potential viral integration against the sequence of the equivalent region. First, for each candidate, this script extracts the host reads with their viral pair from the SAM file of the reads analyzed by ViR_RefineCandidates using the command-line utility “grep” (https://www.gnu.org/software/grep/manual/grep.html); the SAM file is converted into a BAM file using the function “view” of SAMtools [40]; the BAM file is converted into a FASTQ format using the function “bamtofastq” from BEDtools [38] (Fig. 1c, step 1). Then, the script obtains the sequence of the equivalent region in fasta using the BEDtools function “getfasta” [38] (Fig. 1c, step 2). Reads from step 1 are re-aligned to the sequence of the equivalent region using “bwa mem” with default parameters [41]. By taking advantage of the flags of alignment of each read of all chimeric pairs and eventual soft clipped reads, the left and right sides of the potential integration point can be predicted using Trinity (Fig. 1c, step 3). Even if no assemblies are created, flags of alignment are used to predict the direction of the potential integration sites (https://broadinstitute.github.io/picard/explain-flags.html) (Fig. 1c, step 4).

*ViR_LTFinder.* This script is designed to test for an integration from non-host sequences which have a user-defined percentage of similarity to host sequences. WGS reads are mapped to a selected non-host sequence (i.e. an entire genome or selected portions) using “bwa mem” with default parameters [41]. Mapped reads are extracted using the function “view” of SAMtools [40] (Fig. 1d, step 1). The aligned reads are converted into FASTQ format using the function “bamtofastq” from BEDtools [38] (Fig. 1d, step 2) and used for *de-novo* local assembly using Trinity [42] (Fig. 01d, step 3). A consensus sequence is built if any instances of LT are identified. Output of ViR_LTFinder include files for visualization of the aligned reads using the Integrated Genomics Viewer (IGV) tool [43].

### Estimating the gain in solving read dispersion

The utility of ViR in solving read dispersion was assessed using the concept of the ‘Gain Index’ parameter from the Information Theory [37]. This index estimates the weight that each attribute has in building a classification of entities, given n entities each defined through various attributes [37]. The maximization of the Gain index reflects the power of an attribute in segregating entities to different classes. In our case, we used this index to evaluate the gain of enclosing in a single ‘equivalent region’, reads that had been originally assigned to different loci, our classes. The attribute is the “equivalent region’ identified by ViR_SolveDispersion and the entities are all the reads supporting potential integrations (i.e., output of ViR_RefineCandidates).

The gain of running ViR is estimated through the formula:

$$Normalised\,Dispersion\,Gain = \frac{{I - I_{res} }}{I}$$

where *I* is the entropy and ${I}_{res}$ is the residual information.

The entropy *I* is the dispersion of the reads supporting each locus ID in a sample and it is quantified by the formula:

$$I=-\sum_{l}p\left(l\right)*{\mathrm{log}}_{e}p\left(l\right)$$

where $l$ is the locus ID, meaning the host genomic coordinates of the potential integration identified before ViR. $p\left(l\right)$ is the relative frequency of the reads assigned to the locus ID $l$. Entropy is 0 when only one locus ID (i.e., one potential integration site) is identified in the sample (i.e. WGS dataset). Entropy is > 0, when more than one locus ID is identified in the sample.

The residual information ${I}_{res}$ is defined by the formula*:*

$${I}_{res}=-\sum_{g}p\left(g\right)\sum_{l}p\left(l|g\right)*{\mathrm{log}}_{e}p\left(l|g\right)$$

where $g$ is the ID of the equivalent region identified by ViR_SolveDispersion, $p\left(g\right)$ is the relative frequency of the reads in the equivalent region $g$ and $p\left(l|g\right)$ is the relative frequency of reads assigned to the locus ID $l$ in the equivalent region $g$.

The difference between I and ${I}_{res}$ is evaluated for each sample. The ratio of the difference with respect to the initial entropy is calculated to normalize across samples, which were obtained using different experimental set ups (i.e., WGS from single or pools). This operation results in a $Normalised Dispersion Gain$, which ranges between 0 and 1. The closer the value of $Normalised Dispersion Gain$ is to 0, the higher is the gain of ViR in solving the dispersion of reads. To favor intuitive interpretation of results, we show Solve Dispersion Gain as:

$$Solve\,Dispersion\,Gain = 1 - Normalised\,Dispersion\,Gain = 1 - \frac{{I - I_{res} }}{I} = \frac{{I_{res} }}{I}$$

Values of $Solve Dispersion Gain>0$ are found when ViR was able to identify a unique equivalent region for at least two different reads previously assigned to two different loci ID. The higher the value of $Solve Dispersion Gain$, the higher is the performance of ViR.

### Whole-genome sequencing data

Sequencing data of SSMs are as previously described [31]. Sequencing data from pool samples derive from pools of 30 *Ae. albopictus* mosquitoes each. Mosquitoes were collected on the island of La Reunion Island, France in 2017. DNA was extracted using the DNAesy Blood and Tissue Kit following manufacturer’s recommendations (Qiagen, Hilden Germany). DNA-seq library preparation and sequencing on an Illumina HiSeq4000 was performed at Biodiversa srl (Rovereto, Italy).

### Molecular validation of ViR-predicted integration sites

PCR primers were designed on the basis of the potential viral integrations that we identified running ViR on WGS data from *Ae. albopictus* (Additional file 5). These primes were used on DNA extracted from different mosquitoes than those used as source of WGS data. *Aedes albopictus* mosquitoes are reared at the insectary of the University of Pavia as previously described [23]. Genomic DNA was extracted from individual mosquitoes using the Promega Wizard® Genomic DNA Purification Kit, according to manufacturer’s protocol. PCR reactions were carried out with the DreamTaq Green PCR Master Mix (ThermoFisher) using 1 μl of genomic DNA. Amplified bands were purified with ExoSAP-IT kit (ThermoFisher) and send to Macrogen (Madrid, Spain) for Sanger sequencing. Sequences were analyzed with Bioedit [44].

### In silico dataset generation

Several parameters were considered to generate in silico data, including sequencing coverage and viral integration length. A single viral sequence (Dengue virus 2, NCBI reference sequence: NC_001474.2) was selected and portions of different length (300, 600 or 900 bps) of this sequence were in silico integrated in different genomic regions of the *Ae. albopictus* genome, AalbF2 [28]. Three different integration loci were selected among transposon sequences annotated in the genome of the mosquito, based on their frequency: a region present in single copy (UL), a transposon present in 10 copies (Rep10) and a transposon present in 100 copies (Rep100) (Additional file 6). Single samples were simulated at five different average coverages: 5, 15, 30, 45 and 60x. Pool samples were simulated using the same conditions as above (integration loci: UL, Rep10 and Rep100; integration length: 300, 600 and 900 bps) by pooling single sample data, at three different pool coverage, 30, 45 and 60x, and with different pool size (10, 30 and 50 individuals), with the assumption that a single sample carrying a viral integration was introduced in each pool.

Dataset were simulated using WGSIM reads simulator (https://github.com/lh3/wgsim), with read length of 2 × 150 and fragment length of 700 bps. Read number was dynamically adjusted to obtain the desired coverage. The obtained synthetic FastQ were analyzed with Vy-PER, ViR module 1 and ViR module 2. Confusion matrixes were generated, and commonly used performance metrics were calculated (Additional file 3).

## Supplementary Information

**Additional file 1: Viral Database**. List of the viruses and sequences names use to detect novel viral integrations.

**Additional file 2: Vy-PER results in AaloF1 and AalbF2 reference genomes of Aedes albopictus**. The number of reads and the number of clusters detected by Vy-PER are shown in both reference genomes, for each sample.

**Additional file 3: Results of ViR performance using in silico data**. ViR performances were computed based on the confusion matrix, that collects raw counts of correctly and incorrectly detected integration events.

**Additional file 4: Structure of the file “Final_ChimericPairs_Info.txt”**. This is the output of the script ViR _RefineCandidates.

**Additional file 5: PCR Primers**. List of PCR primers used to confirm ViR-predicted viral integrations from WGS data of Aedes albopictus.

**Additional file 6: in silico sequence analysis**. Sequence of the novel viral integrations as detected by ViR.

## Data Availability

The WGS datasets analyzed during the current study are available from the corresponding author on reasonable request. The viral genome database entries are included in the supplementary information file. Dockefile, code and documentations are freely accessible at https://github.com/epischedda/ViR.

## Abbreviations

BAM: Binary alignment map
CPU: Central Processing Unit
EVE: Endogenous viral element
LT: Lateral transfer
RAM: Random access memory
Rep10: Transposon present in 10 copies
Rep100: Transposon present in 100 copies
SAM: Sequence alignment map
TE: Transposable element
UL: Unique locus
WGS: Whole genome sequencing
