# Supplementary material for: ViR: a tool to solve intrasample variability in the prediction of viral integration sites using whole genome sequencing data
Source: BMC Bioinformatics. 2021 Feb 4;22:45. doi: 10.1186/s12859-021-03980-5 (PMC7863434; doi:10.1186/s12859-021-03980-5)
Supplement: Supplementary file 1 — Additional file 1: Viral Database. List of the viruses and sequences names use to detect novel viral integrations. [file 12859_2021_3980_MOESM1_ESM.docx]

**Additional file 1: Viral Database.** List of the viruses and sequences names use to detect novel viral integrations.

| **Viral name** | **Sequence ID** |
| --- | --- |
| Groundnut bud necrosis virus | NC_003620.1, NC_003619.1, NC_003614.1 |
| Murray Valley encephalitis virus | NC_000943.1 |
| Japanese encephalitis virus | NC_001437.1 |
| Venezuelan equine encephalitis virus | NC_001449.1 |
| Dengue virus 2 | NC_001474.2 |
| Dengue virus 3 | NC_001475.2 |
| Dengue virus 1 | NC_001477.1 |
| Onyong-nyong virus | NC_001512.1 |
| Ross River virus | NC_001544.1 |
| Sindbis virus | NC_001547.1 |
| Vesicular stomatitis Indiana virus strain 98COE | NC_001560.1 |
| Tick-borne encephalitis virus | NC_001672.1 |
| Barmah Forest virus | NC_001786.1 |
| Louping ill virus | NC_001809.1 |
| Bunyamwera virus | NC_001927.1, NC_001926.1, NC_001925.1 |
| Yellow fever virus | NC_002031.1 |
| Tomato spotted wilt tospovirus | NC_002052.1, NC_002051.1, NC_002050.1 |
| Dengue virus 4 | NC_002640.1 |
| Semliki Forest virus | NC_003215.1 |
| Mayaro virus | NC_003417.1 |
| Sleeping disease virus | NC_003930.1 |
| Peanut bud necrosis virus | NC_003620.1, NC_003619.1, NC_003614.1 |
| Impatiens necrotic spot virus | NC_003625.1, NC_003624.1, NC_003616.1 |
| Modoc virus | NC_003635.1 |
| Rio Bravo virus | NC_003675.1 |
| Apoi virus | NC_003676.1 |
| Powassan virus | NC_003687.1 |
| Langat virus | NC_003690.1 |
| Eyach virus | NC_003707.1, NC_003706.1, NC_003705.1, NC_003704.1, NC_003703.1, NC_003702.1, NC_003701.1, NC_003700.1, NC_003699.1, NC_003698.1, NC_003697.1, NC_003696.1 |
| Watermelon silver mottle tospovirus | NC_003843.1, NC_003841.1, NC_003832.1 |
| Eastern equine encephalitis virus | NC_003899.1 |
| Aura virus | NC_003900.1 |
| Western equine encephalitis virus | NC_003908.1 |
| Salmon pancreas disease virus | NC_003930.1, NC_003433.1 |
| Tamana bat virus | NC_003996.1 |
| La Crosse virus | NC_004110.1, NC_004109.1, NC_004108.1 |
| Montana myotis leukoencephalitis virus | NC_004119.1 |
| Chikungunya virus | NC_004162.2 |
| Colorado tick fever virus | NC_004191.1, NC_004190.1, NC_004189.1, NC_004188.1, NC_004187.1, NC_004186.1, NC_004185.1, NC_004184.1, NC_004183.1, NC_004182.1, NC_004181.1, NC_004180.1 |
| Yokose virus | NC_005039.1 |
| Omsk hemorrhagic fever virus | NC_005062.1 |
| Uukuniemi virus | NC_005221.1, NC_005220.1, NC_005214.1 |
| Oropouche virus | NC_005777.1, NC_005776.1, NC_005775.1 |
| Palyam virus | NC_005995.1, NC_005994.1, NC_005993.1, NC_005992.1, NC_005991.1, NC_005990.1, NC_005989.1, NC_005988.1, NC_005987.1, NC_005986.1 |
| St Croix River virus | NC_006006.1, NC_006005.1, NC_006004.1, NC_006003.1, NC_006002.1, NC_006001.1, NC_006000.1, NC_005999.1, NC_005998.1, NC_005997.1 |
| African horse sickness virus | NC_006009.1, NC_006019.1, NC_006018.1, NC_006017.1, NC_006016.1, NC_005996.1, NC_006012.1, NC_006021.1, NC_006020.1, NC_006011.1 |
| Bluetongue virus | NC_006013.2, NC_006024.2, NC_006025.1, NC_006023.1, NC_006022.1, NC_006015.1, NC_006014.1, NC_006010.1, NC_006008.1, NC_006007.1 |
| Sandfly fever Naples virus | NC_006319.1, NC_006320.1, NC_006318.1,HM566172.1,HM566171.1,HM566170.1,HM566169.1,HM566168.1,HM566167.1,EF201831.1,EF201830.1,EF201828.1 |
| Thogoto thogotovirus | NC_006508.1, NC_006507.1, NC_006506.1, NC_006504.1, NC_006496.1, NC_006495.1 |
| Usutu virus | NC_006551.1 |
| Getah virus | NC_006558.1 |
| Saint Louis encephalitis virus | NC_007580.2 |
| Yunnan orbivirus | NC_007665.1, NC_007664.1, NC_007663.1, NC_007662.1, NC_007661.1, NC_007660.1, NC_007659.1, NC_007658.1, NC_007657.1, NC_007656.1 |
| Peruvian horse sickness virus | NC_007757.1, NC_007756.1, NC_007755.1, NC_007754.1, NC_007753.1, NC_007752.1, NC_007751.1, NC_007750.1, NC_007749.1, NC_007748.1 |
| Capsicum chlorosis virus | NC_008303.1, NC_008302.1, NC_008301.1 |
| Melon yellow spot virus | NC_008307.1, NC_008306.1, NC_008300.1 |
| Entebbe bat virus | NC_008718.1 |
| Sepik virus | NC_008719.1 |
| Aroa virus | NC_009026.2 |
| Akabane virus | NC_009895.1, NC_009894.1, NC_009896.1 |
| West Nile virus lineage 2 | NC_009942.1, NC_001563.2 |
| Tomato zonate spot virus | NC_010491.1, NC_010490.1, NC_010489.1 |
| Zika virus strain Natal RGN | NC_012532.1, NC_035889.1 |
| Kedougou virus | NC_012533.1 |
| Bagaza virus | NC_012534.1 |
| Highlands J virus | NC_012561.1 |
| Wesselsbron virus | NC_012735.1 |
| Epizootic hemorrhagic disease virus (serotype 1 / strain New Jersey) | NC_013405.1, NC_013404.1, NC_013403.1, NC_013402.1, NC_013401.1, NC_013400.1, NC_013399.1, NC_013398.1, NC_013397.1, NC_013396.1 |
| Fort Morgan virus | NC_013528.1 |
| Rift Valley fever virus | NC_014395.1, NC_014396.1, NC_014397.1 |
| Great Island virus | NC_014524.1, NC_014529.1, NC_014530.1, NC_014531.1, NC_014528.1, NC_014527.1, NC_014526.1, NC_014525.1, NC_014522.1, NC_014523.1 |
| Chandiru virus | NC_015374.1, NC_015373.1, NC_015375.1 |
| Sandfly fever Turkey virus | NC_015413.1, NC_015412.1, NC_015411.1 |
| Aguacate virus | NC_015452.1, NC_015451.1, NC_015450.1 |
| Groundnut ringspot and Tomato chlorotic spot virus reassortant | NC_015469.1, NC_015468.1, NC_015467.1 |
| Tembusu virus | NC_015843.2 |
| Ndumu virus | NC_016959.1 |
| Southern elephant seal virus | NC_016960.1 |
| Whataroa virus | NC_016961.1 |
| Bebaru virus | NC_016962.1 |
| Bean necrotic mosaic virus | NC_018072.1, NC_018071.1, NC_018070.1 |
| SFTS virus HB29 | NC_018138.1, NC_018137.1, NC_018136.1 |
| Aino virus | NC_018465.1, NC_018460.1, NC_018459.1 |
| Sathuperi orthobunyavirus | NC_018466.1, NC_018462.1, NC_018461.1 |
| Shamonda orthobunyavirus | NC_018467.1, NC_018464.1, NC_018463.1 |
| Simbu orthobunyavirus | NC_018478.1, NC_018477.1, NC_018476.1 |
| Ntaya virus | NC_018705.3 |
| Chandipura virus | NC_020805.1 |
| Isfahan virus | NC_020806.1 |
| Wallal virus | NC_022562.1, NC_022561.1, NC_022560.1, NC_022559.1, NC_022558.1, NC_022557.1, NC_022556.1, NC_022555.1, NC_022554.1, NC_022553.1 |
| Mobuck virus | NC_022629.1, NC_022628.1, NC_022627.1, NC_022626.1, NC_022625.1, NC_022624.1, NC_022623.1, NC_022622.1, NC_022621.1, NC_022620.1 |
| Razdan virus | NC_022632.1, NC_022631.1, NC_022630.1 |
| Changuinola virus | NC_022642.1, NC_022641.1, NC_022640.1, NC_022639.1, NC_022638.1, NC_022637.1, NC_022634.1, NC_022636.1, NC_022635.1, NC_022633.1 |
| American bat vesiculovirus TFFN-2013 | NC_022755.1 |
| Tyuleniy virus | NC_023424.1 |
| Kama virus | NC_023439.1 |
| Arumowot virus | NC_023635.1, NC_023634.1, NC_023633.1 |
| Madariaga virus | NC_023812.1 |
| Cat Que virus | NC_024076.1, NC_024075.1, NC_024074.1 |
| Vesicular stomatitis New Jersey virus | NC_024473.1 |
| Heartland virus | NC_024496.1, NC_024495.1, NC_024494.1 |
| Umatilla virus | NC_024510.1, NC_024509.1, NC_024508.1, NC_024507.1, NC_024506.1, NC_024505.1, NC_024504.1, NC_024503.1, NC_024500.1, NC_024499.1 |
| Middelburg virus | NC_024887.1 |
| Maraba virus | NC_025255.1 |
| Vesicular stomatitis Alagoas virus | NC_025353.1 |
| Malpais Spring vesiculovirus | NC_025364.1 |
| Yug Bogdanovac vesiculovirus | NC_025378.1 |
| Perinet vesiculovirus | NC_025394.1 |
| Jutiapa virus | NC_026620.1 |
| Cacipacore virus | NC_026623.1 |
| Sokoluk virus | NC_026624.1 |
| Bhanja virus | NC_027142.1, NC_027141.1, NC_027140.1 |
| Wad Medani virus | NC_027547.1, NC_027546.1, NC_027545.1, NC_027544.1, NC_027543.1, NC_027542.1, NC_027541.1, NC_027540.1, NC_027539.1, NC_027533.1 |
| Chenuda virus | NC_027552.1, NC_027551.1, NC_027550.1, NC_027549.1, NC_027548.1, NC_027538.1, NC_027537.1, NC_027536.1, NC_027535.1, NC_027534.1 |
| Chobar Gorge virus | NC_027562.1, NC_027561.1, NC_027560.1, NC_027559.1, NC_027558.1, NC_027557.1, NC_027556.1, NC_027555.1, NC_027554.1, NC_027553.1 |
| Spanish goat encephalitis virus | NC_027709.1 |
| Chrysanthemum stem necrosis virus | NC_027720.1, NC_027719.1, NC_027718.1 |
| Paraiso Escondido virus | NC_027999.1 |
| Cocal virus | NC_028255.1 |
| Potiskum virus | NC_029054.2 |
| Saboya virus | NC_029054.2, NC_033697.1 |
| Spondweni virus | NC_029055.1 |
| Adana virus | NC_029129.1, NC_029128.1, NC_029127.1 |
| Edge Hill virus | NC_030289.1 |
| Tospovirus kiwifruit/YXW/2014 | NC_030380.1, NC_030379.1, NC_030378.1 |
| Huangpi Tick Virus 2 | NC_031140.1, NC_031139.1, NC_031138.1 |
| Zucchini lethal chlorosis virus | NC_031762.1, NC_031759.1, NC_031758.1 |
| New Mapoon virus | NC_032088.1 |
| Kokobera virus | NC_032088.1, NC_009029.2 |
| Bouboui virus | NC_033693.1 |
| Uganda S virus | NC_033698.1 |
| Jugra virus | NC_033699.1 |
| Meaban virus | NC_033721.1 |
| Gadgets Gully virus | NC_033723.1 |
| Kadam virus | NC_033724.1 |
| Saumarez Reef virus | NC_033726.1 |
| Pepper chlorotic spot virus | NC_033774.1, NC_033773.1, NC_033772.1 |
| Orbivirus SX-2017a | NC_033791.1, NC_033790.1, NC_033789.1, NC_033788.1, NC_033787.1, NC_033786.1, NC_033785.1, NC_033784.1, NC_033783.1, NC_033782.1 |
| Melon severe mosaic tospovirus | NC_033834.1, NC_033833.1, NC_033832.1 |
| Urucuri virus | NC_033843.1, NC_033842.1, NC_033841.1 |
| Ambe virus | NC_033845.1, NC_033844.1, NC_033835.1 |
| Anhanga virus | NC_033846.1, NC_033837.1, NC_033836.1 |
| Munguba virus | NC_033847.1, NC_033831.1, NC_033830.1 |
| Tapara virus | NC_033848.1, NC_033839.1, NC_033838.1 |
| Uriurana virus | NC_033850.1, NC_033849.1, NC_033840.1 |
| Phnom Penh bat virus | NC_034007.1 |
| Yaounde virus | NC_034018.1 |
| Dhori thogotovirus | NC_034263.1, NC_034262.1, NC_034261.1, NC_034256.1, NC_034255.1, NC_034254.1 |
| Oriboca virus | NC_034488.1, NC_034476.1, NC_034475.1 |
| Caraparu virus | NC_034489.1, NC_034478.1, NC_034477.1 |
| Bwamba orthobunyavirus | NC_034490.1, NC_034480.1, NC_034479.1 |
| Nyando virus | NC_034492.1, NC_034491.1, NC_034481.1 |
| Capim virus | NC_034493.1, NC_034483.1, NC_034482.1 |
| Kaeng Khoi virus | NC_034501.1, NC_034500.1, NC_034499.1 |
| Marituba virus | NC_034504.1, NC_034496.1, NC_034495.1 |
| Madrid virus | NC_034505.1, NC_034498.1, NC_034497.1 |
| Guaroa virus | NC_034506.1, NC_034487.1, NC_034486.1 |
| Morreton vesiculovirus | NC_034508.1 |
| Wolkberg virus | NC_034633.1, NC_034632.1, NC_034631.1 |
| Tomato chlorotic spot virus | NC_035484.1, NC_035483.1, NC_035482.1 |
| Kabuto mountain virus | NC_036606.1, NC_036605.1, NC_036604.1 |
| Calla lily chlorotic spot virus | NC_036609.1, NC_036608.1, NC_036607.1 |
| Zerdali virus | NC_037613.1, NC_037612.1, NC_029901.1 |
| Toros virus | NC_037615.1, NC_037614.1, NC_029903.1 |
| Arrabida virus | NC_037617.1, NC_037616.1, NC_029082.1 |
| Iris yellow spot virus | NC_038232.1, NC_029800.1, NC_029799.1 |
| Carajas virus | NC_038285.1 |
| Piry virus | NC_038286.1 |
| Radi vesiculovirus | NC_038287.1 |
| Watermelon bud necrosis virus | NC_038290.1, NC_038289.1, NC_038288.1 |
| Corriparta virus | NC_038573.1, NC_038572.1, NC_038571.1, NC_038570.1, NC_038569.1, NC_038568.1, NC_038567.1, NC_038566.1, NC_038565.1, NC_038564.1 |
| Equine encephalosis virus | NC_038583.1, NC_038582.1, NC_038581.1, NC_038580.1, NC_038579.1, NC_038578.1, NC_038577.1, NC_038576.1, NC_038575.1, NC_038574.1 |
| Eubenangee virus | NC_038593.1, NC_038592.1, NC_038591.1, NC_038590.1, NC_038589.1, NC_038588.1, NC_038587.1, NC_038586.1, NC_038585.1, NC_038584.1 |
| Lebombo virus | NC_038603.1, NC_038602.1, NC_038601.1, NC_038600.1, NC_038599.1, NC_038598.1, NC_038597.1, NC_038596.1, NC_038595.1, NC_038594.1 |
| Orungo virus | NC_038613.1, NC_038612.1, NC_038611.1, NC_038610.1, NC_038609.1, NC_038608.1, NC_038607.1, NC_038606.1, NC_038605.1, NC_038604.1 |
| Warrego virus | NC_038623.1, NC_038622.1, NC_038621.1, NC_038620.1, NC_038619.1, NC_038618.1, NC_038617.1, NC_038616.1, NC_038615.1, NC_038614.1 |
| Cabassou virus | NC_038670.1 |
| Everglades virus | NC_038671.1 |
| Mucambo virus | NC_038672.1 |
| Pixuna virus | NC_038673.1 |
| Rio Negro virus | NC_038674.1 |
| Tonate virus | NC_038675.1 |
| Alajuela virus | NC_038717.1, NC_038716.1, NC_038715.1 |
| Lukuni virus | NC_038720.1, NC_038719.1, NC_038718.1 |
| Anopheles B virus | NC_038721.1 |
| Batama virus | NC_038722.1 |
| Bimiti virus | NC_038725.1, NC_038724.1, NC_038723.1 |
| Catu virus | NC_038728.1, NC_038727.1, NC_038726.1 |
| Gamboa virus | NC_038731.1, NC_038730.1, NC_038729.1 |
| Guajara orthobunyavirus | NC_038734.1, NC_038733.1, NC_038732.1 |
| Guama virus | NC_038737.1, NC_038736.1, NC_038735.1 |
| Kairi virus | NC_038740.1, NC_038739.1, NC_038738.1 |
| Koongol virus | NC_038743.1, NC_038742.1, NC_038741.1 |
| Mosso das Pedras virus | NC_038857.1 |
| Main Drain virus | NC_038943.1, NC_038942.1, NC_038941.1 |
| Tete orthobunyavirus | NC_039185.1, NC_039184.1, NC_039183.1 |
| Wyeomyia orthobunyavirus | NC_039188.1, NC_039187.1, NC_039186.1 |
| Punta Toro virus | NC_039193.1, NC_039192.1, NC_039191.1 |
| Polygonum ringspot tospovirus | NC_039205.1, NC_039204.1, NC_039203.1 |
| Jurona vesiculovirus | NC_039206.1 |
| Kyasanur Forest disease virus | NC_039218.1 |
| Royal Farm virus | NC_039219.1 |
| Kaisodi virus | NC_040494.1, NC_040493.1, NC_040492.1 |
| Oz virus | NC_040735.1, NC_040734.1, NC_040733.1, NC_040732.1, NC_040731.1, NC_040730.1 |
| Alstroemeria yellow spot virus | NC_040743.1, NC_040742.1, NC_040741.1 |
| Rocio virus | NC_040776.1 |
| Ilheus virus | NC_040776.1, NC_009028.2 |
| Guangxi orbivirus | NC_040847.1, NC_040482.1, NC_040481.1, NC_040480.1, NC_040479.1, NC_040478.1, NC_040477.1, NC_040476.1, NC_040475.1, NC_040474.1, |
| Thimiri orthobunyavirus | NC_043034.1 |
| Umbre virus | NC_043037.1, NC_043036.1, NC_043035.1 |
| Banzi virus | NC_043110.1 |
| Bukalasa bat virus | NC_043111.1 |
| Carey Island virus | NC_043112.1 |
| Cowbone Ridge virus | NC_043113.1 |
| Dakar bat virus | NC_043114.1 |
| Israel turkey meningoencephalomyelitis virus | NC_043115.1 |
| Koutango virus | NC_043116.1 |
| Sal Vieja virus | NC_043117.1 |
| San Perlita virus | NC_043118.1 |
| Wongorr virus | NC_043192.1 |
| Frijoles virus VP-161A | NC_043209.1, NC_043208.1, NC_043207.1 |
| Groundnut yellow spot virus | NC_043210.1 |
| Trocara virus | NC_043402.1 |
| Una virus | NC_043403.1 |
| M'Poko virus | NC_043431.1 |
| Groundnut ringspot virus | NC_043503.1, NC_043504.1, NC_043502.1 |
| Mukawa virus | NC_043511.1, NC_043510.1, NC_043509.1 |
| Tensaw virus | NC_043548.1, NC_043547.1, NC_043546.1 |
| Peaton virus | NC_043551.1, NC_043550.1, NC_043549.1 |
| Sabo virus | NC_043554.1, NC_043553.1, NC_043552.1 |
| Sango virus | NC_043557.1, NC_043556.1, NC_043555.1 |
| Jamestown Canyon virus | NC_043560.1, NC_043559.1, NC_043558.1 |
| Leanyer virus | NC_043563.1, NC_043562.1, NC_043561.1 |
| Anhembi virus | NC_043566.1, NC_043565.1, NC_043564.1 |
| Iaco virus | NC_043569.1, NC_043568.1, NC_043567.1 |
| Sororoca virus | NC_043572.1, NC_043571.1, NC_043570.1 |
| Cachoeira Porteira virus | NC_043575.1, NC_043574.1, NC_043573.1 |
| Jatobal virus | NC_043578.1, NC_043577.1, NC_043576.1 |
| Batai virus | NC_043581.1, NC_043580.1, NC_043579.1 |
| Schmallenberg virus | NC_043584.1, NC_043583.1, NC_043582.1 |
| Ilesha virus | NC_043587.1, NC_043586.1, NC_043585.1 |
| Ingwavuma virus | NC_043590.1, NC_043589.1, NC_043588.1 |
| Mermet virus | NC_043593.1, NC_043592.1, NC_043591.1 |
| Utinga virus | NC_043596.1, NC_043595.1, NC_043594.1 |
| Buttonwillow virus | NC_043599.1, NC_043598.1, NC_043597.1 |
| Pacui virus | NC_043602.1, NC_043601.1, NC_043600.1 |
| Rio Preto da Eva virus | NC_043605.1, NC_043604.1, NC_043603.1 |
| Guertu virus | NC_043611.1, NC_043610.1, NC_043609.1 |
| Enseada virus | NC_043614.1, NC_043613.1, NC_043612.1 |
| Fort Sherman virus | NC_043617.1, NC_043616.1, NC_043615.1 |
| Cache Valley virus | NC_043620.1, NC_043619.1, NC_043618.1 |
| Keystone virus | NC_043629.1, NC_043628.1, NC_043627.1 |
| Lumbo virus | NC_043632.1, NC_043631.1, NC_043630.1 |
| Melao virus | NC_043635.1, NC_043634.1, NC_043633.1 |
| San Angelo virus | NC_043638.1, NC_043637.1, NC_043636.1 |
| Serra do Navio virus | NC_043641.1, NC_043640.1, NC_043639.1 |
| Potosi virus | NC_043647.1, NC_043646.1, NC_043645.1 |
| Birao virus | NC_043652.1, NC_043651.1, NC_043650.1 |
| Bozo virus | NC_043655.1, NC_043654.1, NC_043653.1 |
| Laurel Lake virus | NC_043681.1, NC_043680.1, NC_043679.1 |
| Zegla virus | NC_043688.1, NC_043687.1, NC_043686.1 |
| Patois virus | NC_043691.1, NC_043690.1, NC_043689.1 |
| Tacaiuma orthobunyavirus | NC_043694.1, NC_043693.1, NC_043692.1 |
| Shuni orthobunyavirus | NC_043699.1, NC_043698.1, NC_043697.1 |
| Koyama Hill virus | AB894493.1,AB894492.1,AB894491.1,AB894490.1,AB894489.1,AB894488.1,AB894487.1,AB894486.1,AB894485.1,AB894484.1, |
| Cholul virus | JN808310.1,EU879062.3, |
| Salt ash virus | KF234258.1,KF234257.1 |
| El Huayo virus | KT334540.1 |
| Anadyr virus | KU159766.1,KU159765.1,KU159764.1 |
| Parry's Lagoon virus | KU724119.1,KU724118.1,KU724117.1,KU724116.1,KU724115.1,KU724114.1,KU724113.1,KU724112.1,KU724111.1,KU724110.1 |
| Calbertado virus | KX669682.1 |
| Long Pine Key virus | KY290249.1 |
| La Tina virus | KY320649.1 |
| Marisma mosquito virus | MF139576.1 |
| CFLV NEI 1 | M91671.1 |
| Anopheles hinesorum orbivirus | MF073202.1 |
| Anopheles annulipes orbivirus | MF281534.1 |
| Skunk River virus | MK100578.1,MK100577.1,MK100576.1,MK100575.1,MK100574.1,MK100573.1,MK100572.1,MK100571.1,MK100570.1,MK100569.1 |
| Cell fusing agent virus | NC_001564.2 |
| Kamiti River virus | NC_005064.1 |
| Culex flavivirus | NC_008604.2 |
| Quang Binh virus | NC_012671.1, NC_021069.1 |
| Stretch Lagoon orbivirus | NC_012755.1, NC_012754.1 |
| Aedes flavivirus | NC_012932.1 |
| Donggang virus | NC_016997.1 |
| Chaoyang virus | NC_017086.1 |
| Eilat virus | NC_018615.1 |
| Mosquito flavivirus | NC_021069.1 |
| Brazoran virus | NC_022039.1, NC_022038.1, NC_022037.1 |
| Murrumbidgee virus | NC_022597.1, NC_022596.1, NC_022595.1 |
| Fengkai orbivirus | NC_027811.2, NC_027815.1, NC_027814.1, NC_027813.1, NC_027812.1, NC_027807.1, NC_027806.1, NC_027805.1, NC_027804.1, NC_027803.1 |
| Tibet orbivirus | NC_027811.2, NC_027815.1, NC_027814.1, NC_027813.1, NC_027812.1, NC_027807.1, NC_027806.1, NC_027805.1, NC_027804.1, NC_027803.1, |
| Parramatta River virus | NC_027817.1 |
| Mercadeo virus | NC_027819.1 |
| Hanko virus | NC_030401.1 |
| Tai Forest alphavirus | NC_032681.1 |
| Palm Creek virus | NC_033694.1 |
| Nounane virus | NC_033715.1 |
| T'Ho virus | NC_034151.1 |
| Ochlerotatus caspius flavivirus | NC_034242.1 |
| Nanay virus | NC_040610.1 |
| Culex theileri flavivirus | NC_040682.1 |
| Kampung Karu virus | NC_040788.1 |
| Culex pipiens pallens densovirus | EF579756.1 |
| Culex densovirus 0507JS11 | FJ805445.1 |
| Culex Y virus | JQ659255.1,JQ659254.1, |
| Dezidougou virus | JQ675604.1 |
| Santana virus | JQ675606.1 |
| Mosquito X virus | JX403942.1,JX403941.1, |
| Kamphang Phet virus | KC807172.1 |
| Culicine-associated Z virus | KF298272.1,KF298271.1 |
| North Creek virus | KF360973.1,KF360972.1 |
| Mosinovirus | KJ632943.1,KJ632942.1 |
| Wuhan Mosquito Virus 3 | KM817622.1 |
| Wuhan Mosquito Virus 5 | KM817624.1 |
| Wuhan Mosquito Virus 7 | KM817626.1 |
| Jiangxia Mosquito Virus 1 | KM817670.1 |
| Jiangxia Mosquito Virus 2 | KM817671.1 |
| Xinzhou Mosquito Virus | KM817701.1 |
| Qingnian Mosquito Virus | KM817710.1,KM817673.1 |
| Terena virus | KT966493.1 |
| Anopheles Cypovirus | KU169880.1,KU169879.1 |
| Anopheles flavivirus variant 2 | KX148547.1 |
| Anopheles totivirus | KX148550.1 |
| Bolahun virus variant 1 | KX148551.1 |
| Bolahun virus variant 2 | KX148552.1 |
| Gambie virus | KX148553.1 |
| Chaq virus‐like1 | KX148554.1 |
| Chaq virus‐like2 | KX148555.1 |
| Chaq virus‐like3 | KX148556.1 |
| dsRNA virus‐like 1 | KX148558.1 |
| dsRNA virus‐like 2 | KX148559.1 |
| dsRNA virus‐like 3 | KX148560.1 |
| dsRNA virus‐like 4 | KX148561.1 |
| dsRNA virus‐like 5 | KX148562.1 |
| dsRNA virus‐like 6 | KX148563.1 |
| dsRNA virus‐like 7 | KX148564.1 |
| dsRNA virus‐like 8 | KX148565.1 |
| dsRNA virus‐like 9 | KX148566.1 |
| Endornavirus‐like 1 | KX148567.1 |
| Endornavirus‐like 2 | KX148568.1 |
| Endornavirus‐like 3 | KX148569.1 |
| Mononegavirus‐like 1 | KX148570.1 |
| Mononegavirus‐like 2 | KX148571.1 |
| Mononegavirus‐like 3 | KX148572.1 |
| Mononegavirus‐like 4 | KX148573.1 |
| Mononegavirus‐like 5 | KX148574.1 |
| Partitivirus‐like 3 | KX148577.1 |
| ssRNA virus‐like 1 | KX148580.1 |
| ssRNA virus‐like 2 | KX148581.1 |
| ssRNA virus‐like 3 | KX148582.1 |
| ssRNA virus‐like 4 | KX148583.1 |
| ssRNA virus‐like 5 | KX148584.1 |
| ssRNA virus‐like 6 | KX148585.1 |
| Guaico Culex virus | KT966499.2,KM461670.1,KM461669.1,KM461668.1,KM461667.1,KM461666.1 |
| Hubei sobemo‐like virus 39 | KX882828.1 |
| Hubei noda‐like virus 12 | KX883125.1 |
| Hubei partiti‐like virus 22 | KX883195.1 |
| Wuhan Mosquito Virus 4 | KX883867.1,KX883866.1,KX883865.1,KM817623.1 |
| Shuangao partiti‐like virus 1 | KX884100.1 |
| Sinu virus | KX949591.1,KX949590.1,KX949589.1,KX949588.1,KX949587.1,KX949586.1 |
| Panmunjeom flavivirus | KY072986.1 |
| Omono River virus | KY264024.1 |
| Ohlsdorf virus | KY768856.1 |
| Bontang Baru virus | MF158348.1 |
| Culex phasma-like virus | MF176244.1, MF176243.1, MF176242.1 |
| Ngewotan virus | MF176279.1 |
| Aedes alboannulatus reo-like virus | MF176328.1, MF176327.1, MF176326.1, MF176325.1 |
| Shuangao chryso‐like virus 1 | MF176343.1,MF176342.1,MF176341.1,MF176340.1, |
| Wilkie qin-like virus | MF176289.1, MF176290.1, MF176349.1, MF176350.1 |
| Culex mononega-like virus 1 | MF176316.1, MF176317.1 |
| Wuhan Mosquito Virus 6 | MF176383.1,MF176382.1,MF176381.1,MF176380.1,MF176379.1,MF176378.1 |
| Culex Luteo-like virus | MF176386.1, MF176387.1 |
| Hubei chryso‐like virus 1 | MF176391.1,MF176390.1,MF176389.1,MF176388.1 |
| Lobeira virus | MF344595.1,MF344594.1,MF344593.1 |
| Merida‐like Turkey virus | MF882997.1 |
| Barkedji virus | MG214905.1 |
| Houston virus | MH719099.1 |
| Sabethes flavivirus | MH899446.1 |
| Hubei reo‐like virus 7 | MK133924.1 |
| Armigeres subalbatus virus SaX06-AK20 | NC_014609.1 |
| Culex originated Tymoviridae-like virus | NC_018703.1 |
| Anopheline‐associated C virus | NC_023682.1, NC_023683.1, |
| Anopheles minimus iridovirus | NC_023848.1 |
| Tanay virus | NC_024071.1, |
| Mosquito Circovirus | NC_026512.1 |
| Anopheles flavivirus variant 1 | NC_031327.1 |
| Zhejiang mosquito virus 3 | NC_032491.1 |
| Big Cypress virus | NC_034152.1, |
| Biratnagar virus | NC_034153.1, |
| San Bernardo virus | NC_034154.1, |
| Cordoba virus | NC_034156.1, |
| Fort Crockett virus | NC_034157.1, |
| Wilkie Partiti-like virus 2 | NC_035119.1 |
| Wilkie narna-like virus 2 | NC_035120.1 |
| Point Douro narna-like virus | NC_035121.1 |
| Wilkie Partiti-like virus 1 | NC_035122.1 |
| Culex Negev-like virus 2 | NC_035123.1 |
| Culex Negev-like virus 1 | NC_035124.1 |
| Leschenault Partiti-like virus | NC_035125.1 |
| Wilkie narna-like virus 1 | NC_035126.1 |
| Aedes camptorhynchus negev-like virus | NC_035128.1 |
| Culex Negev-like virus 3 | NC_035129.1 |
| Aedes alboannulatus toti-like virus | NC_035130.1 |
| Culex rhabdo-like 1 | NC_035132.1 |
| Culex mononega-like virus 2 | NC_035133.1 |
| Aedes camptorhynchus reo-like virus | NC_035223.1, NC_035222.1, NC_035217.1, NC_035216.1 |
| Bellavista virus | NC_043623.1, NC_043622.1, NC_043621.1 |
| African swine fever virus | NC_044944.1 |
|  |  |
|  |  |
