# Supplementary material for: ViR: a tool to solve intrasample variability in the prediction of viral integration sites using whole genome sequencing data
Source: BMC Bioinformatics. 2021 Feb 4;22:45. doi: 10.1186/s12859-021-03980-5 (PMC7863434; doi:10.1186/s12859-021-03980-5)
Supplement: Supplementary file 2 — Additional file 2: Vy-PER results in AaloF1 and AalbF2 reference genomes of Aedes albopictus. The number of reads and the number of clusters detected by Vy-PER are shown in both reference genomes, for each sample. [file 12859_2021_3980_MOESM2_ESM.docx]

**Additional file 2: Vy-PER results in AaloF1 and AalbF2 reference genomes of *Aedes albopictus*.** The number of reads and the number of clusters detected by Vy-PER are shown in both reference genomes, for each sample.

|  | **AaloF1** | | **AalbF2** | |
| --- | --- | --- | --- | --- |
|  | **Vy-PER hit clusters** | **Vy-PER number of reads** | **Vy-PER hit clusters** | **Vy-PER number of reads** |
| Pool60R1 | 0 | 0 | 0 | 0 |
| Pool60R2 | 108 | 2134 | 36 | 289 |
| Pool60R3 | 0 | 0 | 0 | 0 |
| Pool30R1 | 15 | 36 | 7 | 26 |
| Pool30R2 | 5 | 12 | 10 | 31 |
| Pool30R3 | 18 | 40 | 13 | 39 |
| SSM1 | 50 | 161 | 23 | 73 |
| SSM2 | 27 | 86 | 10 | 29 |
| SSM3 | 0 | 0 | 0 | 0 |
| SSM4 | 25 | 82 | 12 | 43 |
| SSM5 | 28 | 92 | 15 | 43 |
| SSM6 | 44 | 126 | 11 | 29 |
| SSM7 | 27 | 131 | 13 | 87 |
| SSM8 | 37 | 196 | 16 | 123 |
| SSM9 | 37 | 109 | 15 | 36 |
| SSM10 | 34 | 225 | 13 | 37 |
| SSM11 | 0 | 0 | 0 | 0 |
| SSM12 | 27 | 144 | 11 | 97 |
| SSM13 | 0 | 0 | 0 | 0 |
| SSM14 | 27 | 131 | 10 | 79 |
| SSM15 | 0 | 0 | 0 | 0 |
| SSM16 | 39 | 186 | 12 | 109 |
| SSM17 | 40 | 203 | 14 | 129 |
| SSM18 | 35 | 167 | 16 | 115 |
| SSM19 | 39 | 161 | 16 | 132 |
| SSM20 | 39 | 176 | 0 | 0 |
| SSM21 | 0 | 0 | 0 | 0 |
| SSM22 | 27 | 152 | 14 | 96 |
