# Supplementary material for: ViR: a tool to solve intrasample variability in the prediction of viral integration sites using whole genome sequencing data
Source: BMC Bioinformatics. 2021 Feb 4;22:45. doi: 10.1186/s12859-021-03980-5 (PMC7863434; doi:10.1186/s12859-021-03980-5)
Supplement: Supplementary file 3 — Additional file 3: Results of ViR performance using in silico data. ViR performances were computed based on the confusion matrix, that collects raw counts of correctly and incorrectly detected integration events. [file 12859_2021_3980_MOESM3_ESM.docx]

**Additional File 3. Results of ViR performance using *in silico* data.** ViR performances were computed based on the confusion matrix, that collects raw counts of correctly and incorrectly detected integration events.

ViR performance was tested using simulated WGS data from samples sequenced singly (SSM) (A) and sequenced in pools (B). Performance of module 1 and module 2 were tested considering all the *in silico* data produced (‘ALL’) and separately considering five different sequencing coverage depths (cov.): Cov5, Cov15, Cov30, Cov45 and Cov60, and simulating integration events (int. site) in unique genomic loci (UL) or repeated, 10 or 100 times, genomic regions (Rep10 and Rep100). Integration events regard integrations of different sizes (Int. size of 300 bp, 600 bp and 900 bp). For pools, the size of the pool (pools of 10, 30 and 50 individuals) was also tested.

1. SSM Module 1

|  |  | **ACC^1^** | **SENS^2^** | **SPEC^3^** | **PREC^4^** | **F1^5^** | **B ACC^6^** | **MCC^7^** |
| --- | --- | --- | --- | --- | --- | --- | --- | --- |
|  | ALL | 100 | 100 | 100 | 100 | 100 | 100 | 100 |
| **Cov.** | Cov5 | 100 | 100 | 100 | 100 | 100 | 100 | 100 |
|  | Cov15 | 100 | 100 | 100 | 100 | 100 | 100 | 100 |
|  | Cov30 | 100 | 100 | 100 | 100 | 100 | 100 | 100 |
|  | Cov45 | 100 | 100 | 100 | 100 | 100 | 100 | 100 |
|  | Cov60 | 100 | 100 | 100 | 100 | 100 | 100 | 100 |
| **Int. size** | INT0 | 100 | ND | 100 | ND | ND | ND | ND |
|  | 300-INT0 | 100 | 100 | 100 | 100 | 100 | 100 | 100 |
|  | 600-INT0 | 100 | 100 | 100 | 100 | 100 | 100 | 100 |
|  | 900-INT0 | 100 | 100 | 100 | 100 | 100 | 100 | 100 |
| **Int. site** | UL | 100 | 100 | 100 | 100 | 100 | 100 | 100 |
|  | T10 | 100 | 100 | 100 | 100 | 100 | 100 | 100 |
|  | T100 | 100 | 100 | 100 | 100 | 100 | 100 | 100 |

SSM Module 2

|  |  | **ACC^1^** | **SENS^2^** | **SPEC^3^** | **PREC^4^** | **F1^5^** | **B ACC^6^** | **MCC^7^** |
| --- | --- | --- | --- | --- | --- | --- | --- | --- |
|  | ALL | 93,75 | 100 | 78,94 | 91,83 | 95,74 | 89,47 | 85,14 |
| **Cov.** | Cov5 | 100 | 100 | 100 | 100 | 100 | 100 | 100 |
|  | Cov15 | 100 | 100 | 100 | 100 | 100 | 100 | 100 |
|  | Cov30 | 85,71 | 100 | 60 | 81,81 | 90 | 80 | 70,06 |
|  | Cov45 | 92,30 | 100 | 75 | 90 | 94,73 | 87,5 | 82,15 |
|  | Cov60 | 92,30 | 100 | 75 | 90 | 94,73 | 87,5 | 82,15 |
| **Int. size** | INT0 | 100 | ND | 100 | ND | ND | ND | ND |
|  | 300-INT0 | 93,75 | 100 | 88,23 | 88,23 | 93,75 | 94,11 | 88,23 |
|  | 600-INT0 | 93,75 | 100 | 88,23 | 88,23 | 93,75 | 94,11 | 88,23 |
|  | 900-INT0 | 100 | 100 | 100 | 100 | 100 | 100 | 100 |
| **Int. site** | UL | 100 | 100 | 100 | 100 | 100 | 100 | 100 |
|  | T10 | 100 | 100 | 100 | 100 | 100 | 100 | 100 |
|  | T100 | 83,33 | 100 | 55,55 | 78,94 | 88,23 | 77,77 | 66,22 |

1. Pools Module 1

|  |  | **ACC^1^** | **SENS^2^** | **SPEC^3^** | **PREC^4^** | **F1^5^** | **B ACC^6^** | **MCC^7^** |
| --- | --- | --- | --- | --- | --- | --- | --- | --- |
|  | ALL | 65,74 | 54,32 | 100 | 100 | 70,4 | 77,16 | 47,87 |
| **Cov.** | Cov30 | 55,55 | 40,74 | 100 | 100 | 57,89 | 70,37 | 38,29 |
|  | Cov45 | 69,44 | 59,25 | 100 | 100 | 74,41 | 79,62 | 51,63 |
|  | Cov60 | 72,22 | 62,96 | 100 | 100 | 77,27 | 81,48 | 54,61 |
| **Int. size** | INT0 | 100 | ND | 100 | ND | ND | ND | ND |
|  | 300-INT0 | 64,81 | 29,62 | 100 | 100 | 45,71 | 64,81 | 41,70 |
|  | 600-INT0 | 81,48 | 62,96 | 100 | 100 | 77,27 | 81,48 | 67,78 |
|  | 900-INT0 | 85,18 | 70,37 | 100 | 100 | 82,60 | 85,18 | 73,67 |
| **pool size** | POOL10 | 94,44 | 92,59 | 100 | 100 | 96,15 | 96,29 | 87,03 |
|  | POOL30 | 63,88 | 51,85 | 100 | 100 | 68,29 | 75,92 | 46,05 |
|  | POOL50 | 38,88 | 18,51 | 100 | 100 | 31,25 | 59,25 | 23,18 |
| **Int. site** | UL | 80,55 | 74,07 | 100 | 100 | 85,10 | 87,03 | 64,54 |
|  | Rep10 | 61,11 | 48,14 | 100 | 100 | 65 | 74,07 | 43,40 |
|  | Rep100 | 55,55 | 40,74 | 100 | 100 | 57,89 | 70,37 | 38,29 |

Pools Module 2

|  |  | **ACC^1^** | **SENS^2^** | **SPEC^3^** | **PREC^4^** | **F1^5^** | **B ACC^6^** | **MCC^7^** |
| --- | --- | --- | --- | --- | --- | --- | --- | --- |
|  | ALL | 53,33 | 33,33 | 100 | 100 | 50 | 66,66 | 36,11 |
| **Cov.** | Cov30 | 38,70 | 13,63 | 100 | 100 | 24 | 56,81 | 20,93 |
|  | Cov45 | 58,06 | 40,90 | 100 | 100 | 58,06 | 70,45 | 40,90 |
|  | Cov60 | 64,28 | 47,36 | 100 | 100 | 64,28 | 73,68 | 47,36 |
| **Int. size** | INT0 | 100 | ND | 100 | ND | ND | ND | ND |
|  | INT300 | 61,70 | 10 | 100 | 100 | 18,18 | 55 | 24,49 |
|  | INT600 | 78 | 52,17 | 100 | 100 | 68,57 | 76,08 | 60,88 |
|  | INT900 | 72,34 | 35 | 100 | 100 | 51,85 | 67,5 | 48,60 |
| **pool size** | POOL10 | 94,73 | 90 | 100 | 100 | 94,73 | 95 | 90 |
|  | POOL30 | 57,14 | 42,30 | 100 | 100 | 59,45 | 71,15 | 39,83 |
|  | POOL50 | 27,77 | 3,70 | 100 | 100 | 7,14 | 51,85 | 9,75 |
| **Int. site** | UL | 50 | 26,31 | 100 | 100 | 41,66 | 63,15 | 32,08 |
|  | Rep10 | 58,06 | 40,90 | 100 | 100 | 58,06 | 70,45 | 40,90 |
|  | Rep100 | 51,61 | 31,81 | 100 | 100 | 48,27 | 65,90 | 34,54 |

Legend:

Acc^1:^ $Accuracy= \frac{TP+TN}{TP+TN+FN+FP}$

Sens^2^: $Sensitivity= \frac{TP}{TP+FN}$

Spec^3^: $Specificity= \frac{TN}{TN+FP}$

Prec^4^: $Precision= \frac{TP}{TP+FP}$

F1^5^: $F1= \frac{2TP}{2TP+FP+FN}$

B ACC^6^ $: Balanced Accuracy= \frac{Sensitivity+Specificity}{2}$

MCC^7^: $Matthews Correlation Coefficient \left( MCC \right)= \frac{TP\times TN-FP\times FN}{\sqrt{\left( TP+FP \right)\left( TP+FN \right)(TN+FP)(TN+FN)}}$

Where:

TN (True Negative) is the number of events correctly identified as not integration.

FP (False Positive) is the number of integration events erroneously identified.

TP (True Positive) is the number of integration events correctly identified.

FN (False Negative) is the number of integration events missed by the tool
