# Supplementary material for: ViR: a tool to solve intrasample variability in the prediction of viral integration sites using whole genome sequencing data
Source: BMC Bioinformatics. 2021 Feb 4;22:45. doi: 10.1186/s12859-021-03980-5 (PMC7863434; doi:10.1186/s12859-021-03980-5)
Supplement: Supplementary file 4 — Additional file 4: Structure of the file “Final_ChimericPairs_Info.txt”. This is the output of the script ViR _RefineCandidates. [file 12859_2021_3980_MOESM4_ESM.docx]

**Additional file 4.** Structure of the file “Final_ChimericPairs_Info.txt”. This file is the output of the script ViR _RefineCandidates. The file will have 18 columns.

| Column Name | Column Description |
| --- | --- |
| SAMPLE_ID | name of the sample |
| READ_ID | ID of the pair of reads |
| HR_CHR | Chromosome in which the host read is mapped |
| HR_START | Start position of the host read |
| HR_END | End position of the host read |
| HR_SEQ | Sequence of the host read |
| HR_FLAG | Flag of alignment of the host read |
| HR_NT_BQ20 | N. of nt with Phred base quality >20 in the host read |
| HR_MQ | Phred mapping quality of the host read |
| VR_SEQ | Sequence of the viral read |
| VR_FLAG | Flag of alignment of the viral read |
| VR_NT_BQ20 | N. of nt with Phred base quality >20 in the viral read |
| VIRUS_ID | ID and coordinates of the virus mapping to the viral read |
| VIRUS_START |  |
| VIRUS_END |  |
| VIRAL_SEQ | viral sequence matching to the viral read |
| VIRAL_SEQ_LEN | Length of the viral sequence |
| VR_AlignToRef? | Y/N flag of alignment of the viral read within the host genome |
