# Supplementary material for: ViR: a tool to solve intrasample variability in the prediction of viral integration sites using whole genome sequencing data
Source: BMC Bioinformatics. 2021 Feb 4;22:45. doi: 10.1186/s12859-021-03980-5 (PMC7863434; doi:10.1186/s12859-021-03980-5)
Supplement: Supplementary file 5 — Additional file 5: PCR Primers. List of PCR primers used to confirm ViR-predicted viral integrations from WGS data of Aedes albopictus. [file 12859_2021_3980_MOESM5_ESM.docx]

**Additional file 5.** **PCR Primers.** List of PCR primers used to confirm ViR-predicted viral integrations from WGS data of *Aedes albopictus*.

| **nrEVE** | **Primer Sequence** | **F or R** | ***Location*** |
| --- | --- | --- | --- |
|  |  |  |  |
| nrEVEnew-1 | CGACAGCCTGTTCGAATGC | F | *genome* |
| Left Side | GGAACTACGGAAAGGCTGATG | R | nrEVE |
|  |  |  |  |
| nrEVEnew-1 | ATCTGCCACACCCGGGTT | F | *genome* |
| Both sides | GTTGTTCCCCGGGATAACGT | R | *genome* |
|  |  |  |  |
| nrEVEnew-2 | CCGCGCTCTCACTCAGTA | F | *genome* |
| Left Side | ACTGATGATCTTGTGCTGATGG | R | nrEVE |
|  |  |  |  |
| nrEVEnew-6 | AGCGCGGATACATGTCTCAT | F | *genome* |
| Left side | GAAGGATGGGCGAATTCTGG | R | nrEVE |
|  |  |  |  |
| nrEVEnew-4 | CCGCGTTGGTCCCTTCTG | F | nrEVE |
| Right side | TGCTAACGTATAGGGCACTCAC | R | *Genome* |
|  |  |  |  |
| nrEVEnew-4/6 | TTTTCACTCCCGGGACCTG | F | genome |
| Both sides | GTGTTGATCCTTTCCACCGT | R | *nrEVE* |
|  |  |  |  |
| nrEVEnew-5 | CTGGAGAAGCTAAGGGGTCG | F | nrEVE |
| Right side | TTGCCCAAACTCAAGCCACC | R | *Genome* |
|  |  |  |  |
| nrEVEnew-5 | GTATCCGAGGAACACAAGCG | F | nrEVE |
| Both sides | CTACACAACAGCCAACTCCG | R | *genome* |
|  |  |  |  |
| nrEVEnew-7 | TGATTCGATTCTTACAACCCCTA | F | *genome* |
| Both sides | GCGTATAAGCGTGTTGACCT | R | nrEVE |
|  |  |  |  |
| nrEVEnew-8 | AGTACCTTGAGTTGACGGGG | F | nrEVE |
| Right side | GTTCCTGACAGAGTGCGAGA | R | *genome* |
|  |  |  |  |
