# Supplementary material for: ViR: a tool to solve intrasample variability in the prediction of viral integration sites using whole genome sequencing data
Source: BMC Bioinformatics. 2021 Feb 4;22:45. doi: 10.1186/s12859-021-03980-5 (PMC7863434; doi:10.1186/s12859-021-03980-5)
Supplement: Supplementary file 6 — Additional file 6: in silico sequence analysis. Sequence of the novel viral integrations as detected by ViR. [file 12859_2021_3980_MOESM6_ESM.docx]

**Additional file 6: *in silico* sequence analysis.** Sequence of the novel viral integrations as detected by ViR.

Integrated sequence of 300 nt

>NC_001474.2:937-1236 Dengue virus 2, complete genome

ATGCGTTGCATAGGAATGTCAAATAGAGACTTTGTGGAAGGGGTTTCAGGAGGAAGCTGGGTTGACATGTCTTAGAACATGGAAGCTGTGTGACGACGATGGCAAAAAACAAACCAACATTGGATTTTGAACTGATAAAAACAGAAGCCAAACAGCCTGCCACCCTAAGGAAGTACTGTATAGAGGCAAAGCTAACCAACACAACAACAGAATCTCGCTGCCCAACACAAGGGGAACCCAGCCTAAATGAAGAGCAGGACAAAAGGTTCGTCTGCAAACACTCCATGGTAGACAGAGGA

Integrated sequence of 600 nt

>NC_001474.2:4522-5121 Dengue virus 2, complete genome

GCCGGAGTATTGTGGGATGTTCCTTCACCCCCACCCATGGGAAAGGCTGAACTGGAAGATGGAGCCTATAGAATTAAGCAAAAAGGGATTCTTGGATATTCCCAGATCGGAGCCGGAGTTTACAAAGAAGGAACATTCCATACAATGTGGCATGTCACACGTGGCGCTGTTCTAATGCATAAAGGAAAGAGGATTGAACCATCATGGGCGGACGTCAAGAAAGACCTAATATCATATGGAGGAGGCTGGAAGTTAGAAGGAGAATGGAAGGAAGGAGAAGAAGTCCAGGTATTGGCACTGGAGCCTGGAAAAAATCCAAGAGCCGTCCAAACGAAACCTGGTCTTTTCAAAACCAACGCCGGAACAATAGGTGCTGTATCTCTGGACTTTTCTCCTGGAACGTCAGGATCTCCAATTATCGACAAAAAAGGAAAAGTTGTGGGTCTTTATGGTAATGGTGTTGTTACAAGGAGTGGAGCATATGTGAGTGCTATAGCCCAGACTGAAAAAAGCATTGAAGACAACCCAGAGATCGAAGATGACATTTTCCGAAAGAGAAGACTGACCATCATGGACCTCCACCCAGGAGCGGGAAAGACG

Integrated sequence of 900 nt

>NC_001474.2:2422-3321 Dengue virus 2, complete genome

GATAGTGGTTGCGTTGTGAGCTGGAAAAACAAAGAACTGAAATGTGGCAGTGGGATTTTCATCACAGACAACGTGCACACATGGACAGAACAATACAAGTTCCAACCAGAATCCCCTTCAAAACTAGCTTCAGCTATCCAGAAAGCCCATGAAGAGGGCATTTGTGGAATCCGCTCAGTAACAAGACTGGAGAATCTGATGTGGAAACAAATAACACCAGAATTGAATCACATTCTATCAGAAAATGAGGTGAAGTTAACTATTATGACAGGAGACATCAAAGGAATCATGCAGGCAGGAAAACGATCTCTGCGGCCTCAGCCCACTGAGCTGAAGTATTCATGGAAAACATGGGGCAAAGCAAAAATGCTCTCTACAGAGTCTCATAACCAGACCTTTCTCATTGATGGCCCCGAAACAGCAGAATGCCCCAACACAAATAGAGCTTGGAATTCGTTGGAAGTTGAAGACTATGGCTTTGGAGTATTCACCACCAATATATGGCTAAAATTGAAAGAAAAACAGGATGTATTCTGCGACTCAAAACTCATGTCAGCGGCCATAAAAGACAACAGAGCCGTCCATGCCGATATGGGTTATTGGATAGAAAGTGCACTCAATGACACATGGAAGATAGAGAAAGCCTCTTTCATTGAAGTTAAAAACTGCCACTGGCCAAAATCACACACCCTCTGGAGCAATGGAGTGCTAGAAAGTGAGATGATAATTCCAAAGAATCTCGCTGGACCAGTGTCTCAACACAACTATAGACCAGGCTACCATACACAAATAACAGGACCATGGCATCTAGGTAAGCTTGAGATGGACTTTGATTTCTGTGATGGAACAACAGTGGTAGTGACTGAGGACTGCGGAAATAGAGGACCCTCTTTGAGAACA

UL sequence

>NW_021839020.1:40799000-40800000 Aedes albopictus isolate FPA unplaced genomic scaffold, Aalbo_primary.1 scaffold_8

GAGGTTGTACGTTTTCCACAGCTTGGATTGGTTTAAACAACATTTGTCCATAGATCCGGTCAACTTGTTTGCTTCGTGCATGAAATACCAAGCGCATAACGAACGGCGGCATAATATTGAACGTACGTACACAACTCTATCGAACTTACCTCGATTCCACCTTAGGTCTTGGTCATGATCTTCAGATATTCCAGCGAGTTGCGAGCCGCAGCAGTTTGCGCTTCCTTGGCCGTGCTGCCGCTGCCGTGACAAACGGCAACCGGCAACGTCGACAGTTGCACCAAACACTGGAAGCGTCCGGACAGCGTTTTTTTCGTCAATGTCTACGTAGGTCACCTCGAAGCGTTGCTCGGTGGCGATCTCGTGCAACATTTGCACAAAGTCGATCGATTTGTCATTGAGACAAGTGACCTGCGGGGAGGAATGGATGCTTATTAGCAAGCGTGACAGTTTTTTTTTCTGAGGGGTTTTGAATAGCTAGACATAGTTCCAGATAGGAATAAATTAATTTGTTGAATAGGCCTAATTTTGACAGACTCTAACTGTTTTACAAGTATGTACGTCTCTTACCATTCCACCAAAGAGCAGGTCATGATTCCTCTTTCCCACTAACAATAAAGCCATGAAGAAACTTCGATCGGGTCCTGAAACGACTTGCCATTTTGGAACACAGGGGAGAGACTCGCCGGAAATGATTGTCTGAGGATTGACTAGGGTGGGAGTCTCATCAACCCGATCGTTCGACCATGGAAACCAGTATTCGCGTCATTAAAGACGGGCCTCCTATAGCATGCAGTTGAATACTGGATGGCAGGGAGAAATTTTGGGAACGATCTGGAGGCCTAGCTCTATTGTTTCTTTAAAGGCATAGCGGAGCTTGAAAAACGCTCGCCTTTTAGCTGCAATACGAGCATTTCTTAATGGTCTCGTAAAGCTCCTCGTTCATAATCTCAAGAATAGTTTACAATTAGAATGCTTTATTACCTTTGCGAAGCAATCGT

Rep10 sequence

>NW_021838465.1:55657108-55658124 Aedes albopictus isolate FPA unplaced genomic scaffold, Aalbo_primary.1 scaffold_3

TAAGTCGCTTGTCGCTGATGTTGTGGATAATAAGTGGGAGGTCCATATCTTCCTCTTCCACGATTTCCTTGTCCTCTATAATTAGAATATCTGTTGTTTTGTTGGTGGTTACGATGATCAGAAAATCTTGATTGATTATTGAATCGTCCTCTGTTGAATGAATTATGGTATCCTCTGTTTCCATAATTTCTTCCTCGTCCACGGCCCTGATGTGCATACCTGTCATGAGCCATGAAAACTTGTGCTTGTGGAGTTTTATTTTTCTCTCCATTATCACATTCATGTATTTTCTGGGTTGCATCTGTTATTTTTGTGAAAGTCCCTGCTTTCAGTATTAATTTTGTATCATTGTTGGCAATACCGTTAATAAGCGTTTGTACACCCGCTTTTGTAGCAATTTGGTTGGCTTTGTTAGTTGGAATCGTTTCTTCGATGTAGGCTGTTGCCAGACGTAAAGTCAGTTGTTCAACCTGATCGCAAAATGATTCCAGAGAGTCTTTTTGTTTAAGACTTTTCAATTTTGCCAATAAGTTATCAGAGTTGACTTTTGTGGCGCATTGTGTCTTCAGTGTTGTTAATAGATCGTTTAATGTTTGTGCTCCAGTTATTGCTTGCCTAGCATTACTCGTCAGCCTTGCTTTTACCAACTTAAATAAAATTTGATCAGCTGCCGCTTTTTGATCTGGAGTTGCATCTGCAAATTCTTCGTTGACGTTGTCTTTAAATAACGAGACAGCGTCAAGAAATGCGTTGAGATTTTCAGGAGACCCATCGTAAACTTGTACAATGGATGTCCCTAACCTTATGTCCACTTTTGGTGCCATTTTCGCTAACCTACCGCATATAATTATTACTTTTACTAAAGTTTTGAATTTAATCCTTGTCTTGCTTTGACCGGATAATCTTCTACAAATAATTACTGCTTTCCCTAATGTTTTAAATTTTATTTTAGGTCGGTTGGTTTCTAGAATGATGGATTCCTCTAATATTTTGATTGCTGCTTTATATGTTTGTTTT

Rep100 sequence

>NW_021838547.1:212897-213924 Aedes albopictus isolate FPA unplaced genomic scaffold, Aalbo_primary.1 scaffold_373

TATTTTTCATTCAAATTTAATGTTTTCAGTGTCAAATGTACTAAATTCCAAAGTTCACTATAAATGTTTTGCGATATTCAATGTCACCATTCGAAATGACATGCACTTCAAACCCGCATACAACTTGCATGCAAGTCGCCTTCACAAGTCTAATACATGTAAATAAAATTCCTGCCGTTTTATCCACTACACCACGAAACCCGAACAATCTCGAGGTTCAATGTTCAAGCCCGAACTCTCTCGATAAAATTGGATCGGGTATTTAAAAATAGATGGAGCGATACAGATCATTTAGATGGGTATGAAAGTGAAAGAGATAGCAATTTATTCGGTTCTCATTCGTGGTTGATGAGAATTGTCGTTTGAAAAGATCGTAAAATTACTTCGGTAATTTTACGTATTTCCGGCCACGAAGTCTTGGAATCCGACCTTATCTGAGGACTAACAGAACTTCTACTCGTCCATTTTGACCTGGACCGTGGAAGAGGCGGAAGTGCGTGTTAAAGTTCTTGTGTCGCGGTGTGTTTAATTAAGTATGTTCTTTAAAACGTGAGAAGTCTTTAGATTGAATTAATGTTACATTTTGTTCCTTTCCGTTAGTTAGCCAGTTCAATAACCTAAGCCAAAAGGTTAAGTGCGGTCAAGTAGATTGTGTATGCGTGTTAGTGAATAGAAAAAGGTAATTGATATTTTTTGTTGTGTGGAGTAAACTTTTTGTTAAATGTGTCTAACAGCCGTTCGACGGCTTTTTATTCAGGATTTCGATCTAGCAATCCACGGCCGAAAGCTCGGCACACCACACCTTTTTCGCTAAACGCGCTCTTGACCCCACGTCGCAGCGTCAAAAGTCCCGGTTTGAGGGGAAAGCCCGAAGGGAACAACCGTTCCGACAGAGCGGCCGAAACCACGTTTTCCTTCGGCGTTCATGTGGTAGCCACCACACCGACCAAGCCATCGACGACCCCCGTCGCCCTCAACATCAACAAGCAACTGCGCCGGTAATCCGGTCCGTTTAGCGACCGGCAGCG
